# Validation and optimization of two models for the magnetic restoring forces using a multi-stable piezoelectric energy harvester

**DOI:** 10.1177/1045389X221151064

**Published:** 2023-01-21

**Authors:** Haining Li, Kefu Liu, Jian Deng, Bing Li

**Affiliations:** 1 Department of Mechanical Engineering, Lakehead University, Thunder Bay, ON, Canada; 2 Department of Civil Engineering, Lakehead University, Thunder Bay, ON, Canada; 3 College of Mechanical and Electrical Engineering, Harbin Institute of Technology, Shenzhen, Guangdong, China

**Keywords:** Multi-stable energy harvester, magnetic restoring force, equivalent magnetic point dipole approach, equivalent magnetic 2-point dipole approach, model validation and optimization

## Abstract

This article presents a tunable multi-stable piezoelectric energy harvester. The apparatus consists of a stationary magnet and a cantilever beam whose free end is attached by an assembly of two cylindrical magnets that can be moved along the beam and a small cylindrical magnet that is fixed at the beam tip. By varying two parameters, the system can assume three stability states: tri-stable, bi-stable, and mono-stable, respectively. The developed apparatus is used to validate two models for the magnetic restoring force: the equivalent magnetic point dipole approach and the equivalent magnetic 2-point dipole approach. The study focuses on comparing the accuracy of the two models for a wide range of the tuning parameters. The restoring forces of the apparatus are determined dynamically and compared with their analytical counterparts based on each of the models. To improve the model accuracy, a model optimization is carried out by using the multi-population genetic algorithm. With the optimum models, the parametric sensitivity of each of the models is investigated. The stability state region is generated by using the optimum second model.

## 1. Introduction

In this century, wireless sensor networks have been playing an important role in the Internet of Things (IoT). Normally, the wireless sensors are powered by batteries which are not eco-friendly. It has been much desirable to use vibration energy harvesters to solve costly battery replacement problem and make wireless sensor networks autonomous (Tian et al., 2018). Ambient vibration can be converted to electricity by four methods: piezoelectric (Derayatifar et al., 2021; Lu et al., 2018), electromagnetic (Halim et al., 2015; Pan et al., 2019; Williams and Yates, 1996; Zaghari et al., 2019), electrostatic (Choi et al., 2011; Koga et al., 2017; Zhang et al., 2018), and triboelectric (Tao et al., 2020; Wang et al., 2018b). The main advantages of the piezoelectric vibration energy harvesters (PVEHs) are their large power densities and ease of operation.

A traditional PVEH is a single-degree-of-freedom linear oscillator that performs efficiently only at resonance (Roundy and Wright, 2004; Roundy et al., 2003; Yu and He, 2013). To broaden the response frequency bandwidth, various nonlinear energy harvesters have been proposed (Ramlan et al., 2010; Twiefel and Westermann, 2013; Yang et al., 2018, 2019). According to the system stability, the nonlinear PVEHs can be classified as mono-stable and multi-stable, such as bi-stable or tri-stable. The nonlinearity can be realized by introducing the nonlinear restoring forces to the piezoelectric beam. Applying magnetic forces to the beam is one of the convenient ways to achieve that. Stanton et al. (2009) reported a PVEH that consists of a piezoelectric cantilever beam with a tip magnet subjected to an external magnetic field generated by a pair of fixed magnets. Such a mono-stable energy harvester can exhibit softening or hardening behaviors when the magnetic interaction is adjusted. By applying different external magnet tuning strategies, two kinds of bi-stable energy harvester (BEH) can be achieved: attractive magnetic force type (Fu and Yeatman, 2019; Rezaei et al., 2021) or repulsive magnetic force type (Bae and Kim, 2021; Wang et al., 2018a; Xie et al., 2019). Zhao and Erturk (2013) found that for a certain range of excitation intensity, the BEH can largely enhance the power output performance due to its snap-through characteristic. Further, in order to reduce the potential barrier of BEHs, tri-stable energy harvesters (TEH) have been proposed by introducing a middle potential well between BEH’s two potential wells. Based on the configuration of the BEH proposed by Erturk et al. (2009), TEHs were achieved by tuning the angular orientations (Cao et al., 2015; Zhou et al., 2013) or the spatial positions (Haitao et al., 2016; Wang et al., 2019) of the two fixed magnets. However, the disadvantage of the aforementioned tunable multi-stable PVEHs is that they need more than one fixed magnet to achieve the tri-stable state. Thus, installing multiple fixed magnets would take more space, which is undesirable in realization through a micro-electromechanical system (MEMS). And also, an asynchronous operation of tuning the angle or position of the fixed magnets will lead to the asymmetric potential wells for the TEH.

In the dynamic modeling of a tunable multi-stable PVEH, an accurate magnetic force model is crucial. Generally, the magnetic force between two magnets is complicated, especially when the separation distance between them is relatively small. There are several commonly used models for this purpose. The most widely used one is the so-called equivalent magnetic point dipole approach (Fang et al., 2019; Stanton et al., 2010; Tang and Yang, 2012) which treats each magnet as a point dipole at its center. However, this approach has a limitation as it can offer a reliable prediction only when the distance between the magnets is much greater than their dimensions. In light of this limitation, a magnetic force modeling method based on the equivalent magnetizing current theory was proposed (Leng et al., 2017; Tan et al., 2015). The study indicates that the magnetizing current model offers better accuracy than the equivalent magnetic point dipole model. Accordingly, Wang et al. (2020) proposed an equivalent magnetic 2-point dipole approach, which only counts the magnetizing current on the permanent magnet’s left and right polarized surfaces and uses two total surface charges to represent a magnet. It has been proved that the accuracy of the equivalent magnetic 2-point dipole model was significantly improved by using the proposed method (Ju et al., 2021; Rezaei et al., 2021; Wang et al., 2020; Zhang et al., 2021). Currently, such approach is mainly used in the modeling of the magnetic force between thin cubic permanent magnets, the accuracy of the magnetic force model of the thick cylinder magnets based on such approach still needs to be examined.

In this study, a new tunable multi-stable piezoelectric energy harvester is proposed. Different from the existing design which employs multiple fixed magnets to achieve a tri-stable state, the proposed apparatus consists of a stationary magnet and a cantilever beam whose free end is attached by an assembly of two cylindrical magnets that can be moved along the beam and a small cylindrical magnet that is fixed at the beam tip. By varying the gap between the stationary magnet and the tip magnet, and the distance between the magnet assembly and the tip magnet, the system can assume three stability states: tri-stable, bi-stable, and mono-stable, respectively. Modeling the magnetic restoring forces for a tunable multi-stable energy harvester poses a challenge as a reliable model should give an accurate prediction over a wide range of the tuning parameters. For this purpose, the developed apparatus is used to dynamically validate two commonly used models: the equivalent magnetic point dipole approach and the equivalent magnetic 2-point dipole approach proposed by Wang et al. (2020). The study shows that although the second model offers more accurate results than the first model, it still fails to predict the restoring forces in some cases. A numerical optimization is carried out to improve the accuracy of both models. The study shows that by using the optimum parameters, both models can achieve a comparable accuracy.

The rest of the paper is organized as follows. Section 2 presents the proposed apparatus. Section 3 derives the magnetic restoring force models based on the equivalent magnetic point dipole approach and the equivalent magnetic 2-point dipole approach, respectively. Section 4 validates the two models dynamically. Section 5 conducts a model optimization. Section 6 uses the optimum model for the parametric sensitivity study and the stability region determination. Finally, Section 7 draws the main conclusions of the study.

## 2. Apparatus

**Figure 1.**
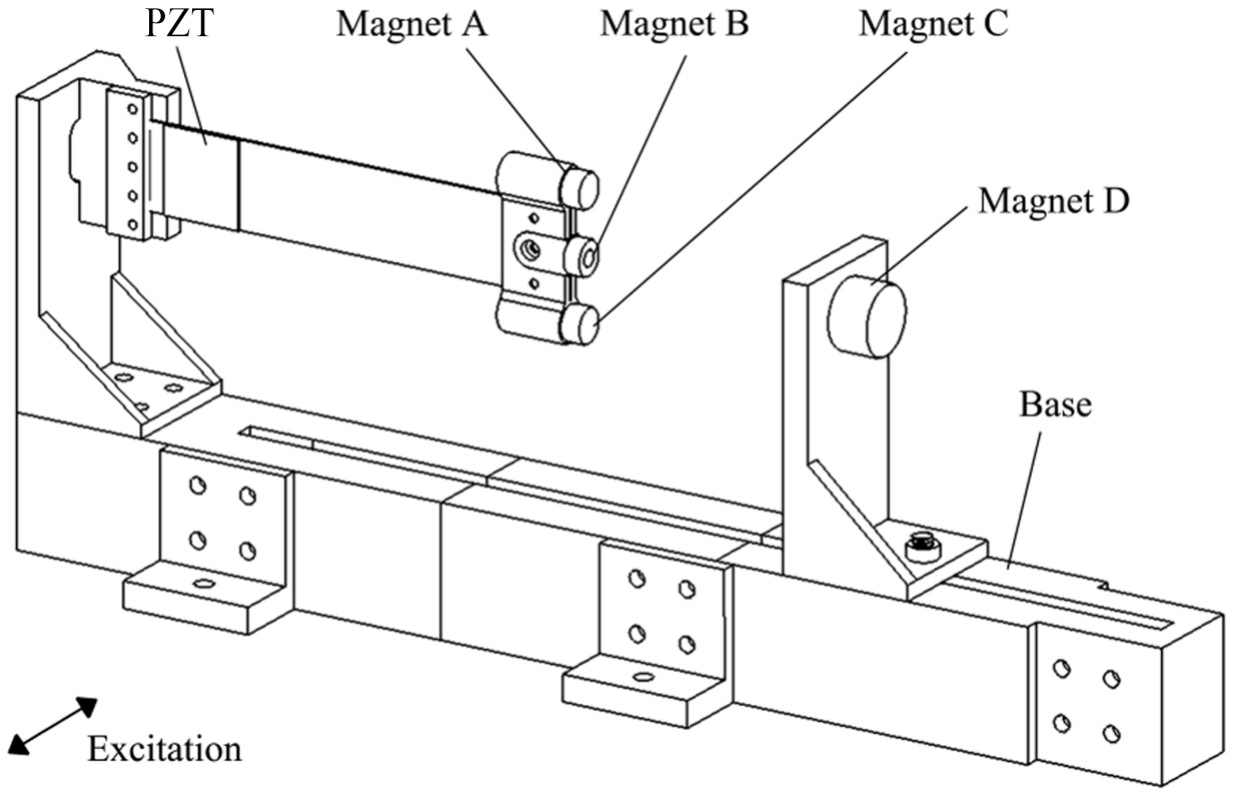
Schematic of the apparatus.

**Figure 2.**
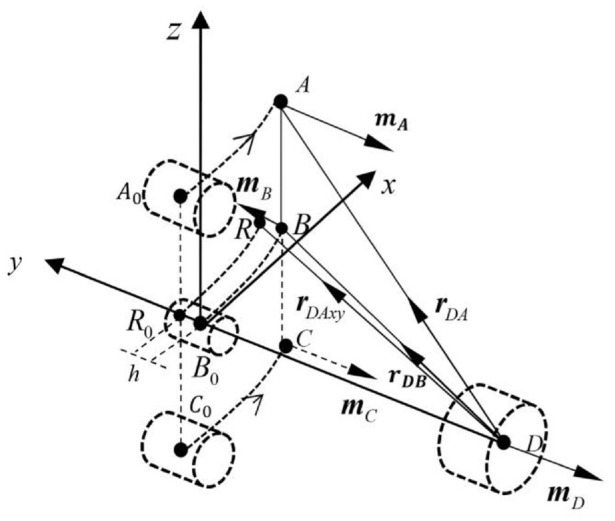
Spatial positions of the magnets.

Figure 1 shows a CAD drawing of the developed apparatus. A cantilever beam is constructed by connecting a piezoelectric transducer (S128-J1FR-1808YB, Midé) to a thin stainless-steel plate. One end of the cantilever beam is clamped to a stand that is fastened to a base, while its other end is fixed with a small cylindrical magnet B and attached with a holder for an assembly of two identical cylindrical magnets A and C. The holder for magnets A and C can slide along the beam. A large cylindrical magnet D is fixed in a stand that can slide along the base. When the cantilever beam is at its equilibrium position or undeflected, the four magnets situate on the same vertical plane and magnets B and D are collinear. By sliding the stand for magnet D, the distance between magnet B and magnet D can be adjusted. By sliding the holder along the beam, the distance between magnet B and magnets A, C can be varied. Figure 2 illustrates the spatial positions and polarities of the four magnets where $m_{A}$ , $m_{B}$ , $m_{C}$ , $m_{D}$ are the magnetic moment vectors, $A_{0}$ , $\;B_{0}$ , $\;C_{0}$ and *A*, *B*, *C* denote the center positions of magnets A, B, and C when the beam is undeformed and deformed, respectively, and the origin of the coordinate system is also located at $B_{0}$ , $r_{\mathrm{DA}}$ represents a vector from *A* to *D*, $r_{\mathrm{DB}}$ represents a vector from *B* to *D*, and the vector $r_{\mathrm{DA}}$ ’s projection on the *x*-*y* plane is represented by $r_{\mathrm{DAxy}}$ .

**Figure 3.**
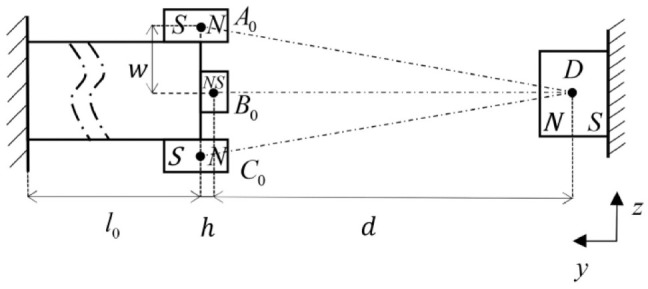
Front view of the apparatus.

**Figure 4.**
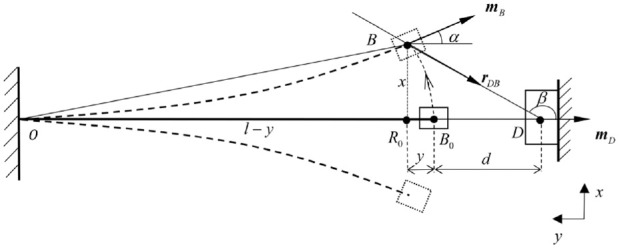
Top view of the apparatus.

Figures 3 and 4 show the front view and top view of Figure 2, respectively, where *d* is the distance between magnet D and magnet B when the beam is undeformed, and h is the distance between magnet A or C and magnet B, *l* is the length of the cantilever beam, and *w* is the distance between the axis of magnet B and that of magnet A or C.

## 3. The restoring force of the system

The total restoring force $f_{x}$ of the system in the *x*-direction consists of a restoring force $f_{e}$ due to the beam’s elasticity, an attractive magnetic force $f_{\mathrm{DBx}}$ between magnet D and magnet B and two repulsive magnetic forces $f_{\mathrm{DAx}}$ between magnet D and magnet A, and $f_{\mathrm{DCx}}$ between magnet D and magnet C. Since magnets A and C are identical and symmetrical about the central line of the beam, the values of $f_{\mathrm{DAx}}$ and $f_{\mathrm{DCx}}$ are equal. Then the total restoring force can be expressed as:

(1) $$f_{x}=f_{e}+f_{\mathrm{DBx}}+f_{\mathrm{DAx}}+f_{\mathrm{DCx}}=k_{b}x+f_{\mathrm{DBx}}+2f_{\mathrm{DAx}}$$

where $k_{b}$ is the stiffness of the beam which can be determined experimentally. In what follows, the analytical restoring forces $f_{\mathrm{DBx}}$ and $f_{\mathrm{DAx}}$ will be found using two approaches.

### 3.1. Equivalent magnetic point dipole model

Commonly, a pair of magnets is regarded as equivalent magnetic point dipoles by assuming that the magnet sizes are much smaller than their separation distance (Yung et al., 1998). Firstly, the magnetic force between magnet B and magnet D is considered. According to this approach, the force exerted by magnet B on magnet D is given by:

(2) $$f_{\mathrm{DB}}=\nabla \left(B_{\mathrm{DB}}\cdot m_{B}\right)$$

where ∇ denotes the vector gradient operator and $B_{\mathrm{DB}}$ is the magnetic flux density generated by magnet D upon B. Equation (2) can be expanded as:

(3) $$\begin{array}{l} f_{DB}=\frac{3\mu_{0}m_{D}m_{B}}{4\pi r_{DB}^{4}}[{\hat{r}}_{DB}\left({\hat{m}}_{B}\cdot {\hat{m}}_{D}\right)-5{\hat{r}}_{DB}\left({\hat{m}}_{D}\cdot {\hat{r}}_{DB}\right)\left({\hat{r}}_{DB}\cdot {\hat{m}}_{B}\right)\\ +{\hat{m}}_{B}\left({\hat{m}}_{D}\cdot {\hat{r}}_{DB}\right)+{\hat{m}}_{D}\left({\hat{m}}_{B}\cdot {\hat{r}}_{DB}\right)] \end{array}$$

where $m_{B}$ , $m_{D}$ , and $r_{\mathrm{BD}}$ are the magnitude of $m_{B}$ , $m_{D}$ , and $r_{\mathrm{DB}}$ , respectively, ${\hat{m}}_{B}$ , ${\hat{m}}_{D}$ , and ${\hat{r}}_{\mathrm{DB}}$ are the unit vector of $m_{B}$ , $m_{D}$ , and $r_{\mathrm{DB}}$ , respectively. These unit vectors can be expressed as:

(4) $$\begin{array}{c} {\hat{m}}_{B}=\left[\sin(\alpha )\;-\cos(\beta )\;0\right],\;{\hat{m}}_{D}=\left[0\;-1\;0\right],\;\\ {\hat{r}}_{\mathrm{DB}}=\left[-\sin(\beta )\;\cos(\beta )\;0\right]. \end{array}$$

Substituting the above unit vectors in equation (3) and the magnetic force in the *x*-direction can be obtained in the following form:

(5) $$\begin{array}{c} f_{\mathrm{DBx}}=-\frac{3\mu_{0}m_{D}m_{B}}{4\pi r_{\mathrm{BD}}^{4}}\\ \;\{\sin\left(\beta \right)\left[\cos\left(\alpha \right)-5\cos\left(\beta \right)\cos\left(\beta -\alpha \right)\right]+\sin(\alpha )\cos\left(\beta \right)\}. \end{array}$$

Since the slope of the beam’s tip is relatively small, it is assumed that $∠\mathrm{BO}B_{0}\;\approx \;\alpha$ . Also, it is noted that β can be found from the triangle $DR_{0}B$ . Thus, equation (5) can be expressed as follows:

(6) $$\begin{array}{c} f_{\mathrm{DBx}}=-\frac{3\mu_{0}m_{D}m_{B}x}{4\pi r_{\mathrm{DB}}^{5}l}\\ \;\{l-2y-d-\frac{5}{r_{\mathrm{DB}}^{2}}[-y^{3}+\left(l-2d\right)y^{2}+\left(2\mathrm{dl}-d^{2}\right)y+d^{2}l-\left(y+d\right)x^{2}]\} \end{array}$$

where $\;y\;=\;l-\sqrt{l^{2}-x^{2}}$ . Similarly, the magnetic force between magnet A and magnet D in the *x*-direction can be found as:

(7) $$\begin{array}{l} f_{DAx}=\frac{3\mu_{0}m_{D}m_{A}x}{4\pi r_{DA}^{4}l_{0}}\{\frac{l_{0}-2y-d_{0}}{r_{DAxy}}-\frac{5}{r_{DAxy}^{3}}[-y^{3}+\left(l_{0}-2d_{0}\right)y^{2}\\ +\left(2d_{0}l_{0}-{d_{0}}^{2}\right)y+{d_{0}}^{2}l_{0}-\left(y+d_{0}\right)x^{2}]\} \end{array}$$

where $d_{0}\;=\;d\;+\;h$ and $l_{0}\;=\;l-h$ . Substituting equations (6) and (7) into equation (1) yields the analytical model of the total restoring force. In equations (6) and (7), the magnitudes of the magnetic moment vectors are determined by:

(8) $$m_{A}=MV_{A},m_{B}=MV_{B},m_{D}=MV_{D},$$

where $V_{A}$ , $V_{B}$ , and $V_{D}$ are the volume of the magnets A, B, and D, respectively, $M=B_{r}/\mu$ is the magnetization of magnets A, B, and D, where $B_{r}\;=\;1.46\;T\;\mathrm{is}\;\mathrm{the}\;\mathrm{magnetic}\;\mathrm{residual}\;\mathrm{flux}\;\mathrm{density}\;\mathrm{and}$ $\mu \;=\;4\pi \times 10^{-7}H/m$ is the vacuum permeability.

### 3.2. Equivalent magnetic 2-point dipole model

**Figure 5.**
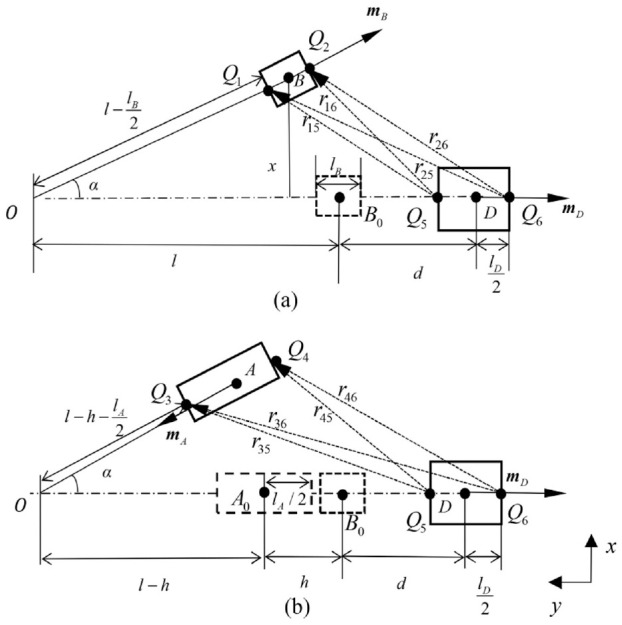
Top view of positions of magnets of the apparatus: (a) magnet B and D and (b) magnet A and D.

As Leng et al. (2017) mentioned, the equivalent magnetic point dipole approach’s accuracy deteriorates when the separation space between the magnets becomes small. In light of such limitation, an improved approach was proposed by Wang et al. (2020). In this study, such an improved approach is named as equivalent magnetic 2-point dipole model as the approach treats a magnet as a 2-point dipole. In what follows, the magnetic restoring force of the system is developed using this improved method. Figure 5(a) and (b) show the top view of the apparatus when the beam is deformed.

As shown in Figure 5(a) and (b), the origin of the coordinate system locates at $B_{0}$ , the centers of the magnet A, B, and D are represented by points *A*, *B*, and *D*, respectively, point $A_{0}$ and $B_{0}$ depict the positions of the magnets A and B when the beam is undeformed, $l_{B}$ , $l_{D}$ , and $l_{A}$ are the length of magnet B, magnet D, and magnet A, respectively, and h is the distance between magnet A or C and magnet B in *y*-direction, *l* is the length of the cantilever beam, $r_{15},\;r_{25},\;r_{35}$ , and $r_{45}$ are the vectors from $Q_{5}$ to $Q_{1},\;Q_{2},\;Q_{3}$ , and $Q_{4}$ , respectively, and $r_{16},\;r_{26},\;r_{36}$ , and $r_{46}$ are the vectors from $Q_{6}$ to $Q_{1},\;Q_{2},\;Q_{3}$ , and $Q_{4}$ respectively, $Q_{1}\;\mathrm{and}\;Q_{2}$ , are the total charges of the left and the right surfaces of magnet B, respectively, $Q_{3}$ and $Q_{4}$ are the total charges of the left and the right surfaces of magnet A, respectively, $Q_{5}$ and $Q_{6}$ are the total charges of the left and right surfaces of magnet D, respectively. The total surface charges can be expressed as follow:

(9) $$\begin{array}{c} Q_{1}=-MS_{B},\;Q_{2}=MS_{B},\;Q_{3}=MS_{A},\;Q_{4}=-MS_{A}\\ Q_{5}=-MS_{D},\;Q_{6}=MS_{D} \end{array}$$

where $S_{B}$ , $S_{A}$ , and $S_{D}$ are the surface area of magnets B, A, and D, respectively, and M is the magnetization of the magnets defined previously.

Similar to the previous section, the magnetic force between magnet B and magnet D is considered first. Based on the Boit-Savart law, the magnetic force exerted by magnet B on magnet D is the combination of the magnetic force exerted from $Q_{1}$ and $Q_{2}$ to $Q_{5}$ and $Q_{6}$ , which is given in the following equation:

(10) $$f_{\mathrm{DB}}=B_{1}Q_{1}+B_{2}Q_{2}$$

where $B_{1}$ and $B_{2}$ are the magnetic current density at $Q_{1}$ and $Q_{2}$ generated by $Q_{5}$ and $Q_{6}$ which can be defined as follows:

(11) $$\begin{array}{c} B_{1}=\frac{\mu_{0}}{4\pi }\left(Q_{5}\frac{X_{5}-X_{1}}{{\left|X_{5}-X_{1}\right|}^{3}}+Q_{6}\frac{X_{6}-X_{1}}{{\left|X_{6}-X_{1}\right|}^{3}}\right),\\ B_{2}=\frac{\mu_{0}}{4\pi }\left(Q_{5}\frac{X_{5}-X_{2}}{{\left|X_{5}-X_{2}\right|}^{3}}+Q_{6}\frac{X_{6}-X_{2}}{{\left|X_{6}-X_{2}\right|}^{3}}\right) \end{array}$$

were $X_{1}$ , $X_{1}$ , $X_{5}$ , and $X_{6}$ are the position vectors of $Q_{1}$ , $Q_{2}$ , $Q_{5}$ , and $Q_{6}$ , respectively, and they can be obtained from Figure 5(a):

(12) $$\begin{array}{c} X_{1}=\left(x-\frac{l_{B}}{2}\sin\alpha \right)i+\left[l-\left(l-\frac{l_{B}}{2}\right)\cos\alpha \right]j,\\ X_{2}=\left(x+\frac{l_{B}}{2}\sin\alpha \right)i+\left[l-\left(l+\frac{l_{B}}{2}\right)\cos\alpha \right]j,\\ X_{5}=-\left(d-\frac{l_{D}}{2}\right)j,X_{6}=-\left(d+\frac{l_{D}}{2}\right)j \end{array}$$

where ***i*** and ***j*** are the unit vector on *x* and *y*-axis. By substituting equations (11) and (12) into equation (10), the magnetic force between magnet B and magnet D can be obtained. It should be noted that the total magnetic force between magnet B and magnet D can be separated into two components: one is in the *y*-direction $f_{\mathrm{DBy}}j$ , another one is in the *x*-direction $f_{\mathrm{DBx}}i$ . According to equation (1), to obtain the total restoring force, only the $f_{\mathrm{DBx}}$ is considered, which can be expressed as follows:

(13) $$f_{\mathrm{DBx}}=-\frac{\mu_{0}}{4\pi }\{Q_{1}\left[Q_{5}\frac{\left(x-\frac{l_{B}}{2}\sin\alpha \right)}{\gamma_{1}}+Q_{6}\frac{\left(x-\frac{l_{B}}{2}\sin\alpha \right)}{\gamma_{2}}\right]\;+Q_{2}\left[Q_{5}\frac{\left(x+\frac{l_{B}}{2}\sin\alpha \right)}{\gamma_{3}}+Q_{6}\frac{\left(x+\frac{l_{B}}{2}\sin\alpha \right)}{\gamma_{3}}\right]\}$$

where $\gamma_{1}$ , $\gamma_{2}$ , $\gamma_{3}$ , and $\gamma_{4}$ can be expressed as follows:

(14) $$\begin{array}{c} \gamma_{1}=\{{\left\{-\left[d-\frac{l_{D}}{2}\right]-\left(l-\left(l-\frac{l_{B}}{2}\right)\cos\alpha \right)\right\}}^{2}+{\left(x-\frac{l_{B}}{2}\sin\alpha \right)}^{2}{\}}^{3/2} \end{array}$$

(15) $$\begin{array}{c} \gamma_{2}=\{{\left\{-\left[d+\frac{l_{D}}{2}\right]-\left(l-\left(l-\frac{l_{B}}{2}\right)\cos\alpha \right)\right\}}^{2}+{\left(x-\frac{l_{B}}{2}\sin\alpha \right)}^{2}{\}}^{3/2} \end{array}$$

(16) $$\begin{array}{c} \gamma_{3}=\{{\left\{-\left[d-\frac{l_{D}}{2}\right]-\left(l-\left(l+\frac{l_{B}}{2}\right)\cos\alpha \right)\right\}}^{2}+{\left(x+\frac{l_{B}}{2}\sin\alpha \right)}^{2}{\}}^{3/2} \end{array}$$

(17) $$\begin{array}{c} \gamma_{4}=\{{\left\{-\left[d+\frac{l_{D}}{2}\right]-\left(l-\left(l+\frac{l_{B}}{2}\right)\cos\alpha \right)\right\}}^{2}+{\left(x+\frac{l_{B}}{2}\sin\alpha \right)}^{2}{\}}^{3/2} \end{array}$$

By following the same process, the magnetic force between magnet A and D in the *x*-direction can also be obtained as:

(18) $$f_{\mathrm{DAx}}=-\frac{\mu_{0}}{4\pi }\{Q_{3}\left[Q_{5}\frac{\left(x-h\sin\alpha -\frac{l_{A}}{2}\right)}{\gamma_{5}}+Q_{6}\frac{\left(x-h\sin\alpha -\frac{l_{A}}{2}\right)}{\gamma_{6}}\right]+Q_{4}\left[Q_{5}\frac{\left(x-h\sin\alpha +\frac{l_{A}}{2}\right)}{\gamma_{7}}+Q_{6}\frac{\left(x-h\sin\alpha +\frac{l_{A}}{2}\right)}{\gamma_{8}}\right]\}$$

where $\gamma_{5}$ , $\gamma_{6}$ , $\gamma_{7}$ , and $\gamma_{8}$ can be expressed as follows:

(19) $$\gamma_{5}=\{{\left\{-\left[d-\frac{l_{D}}{2}\right]-\left(l-\left(l-h-\frac{l_{A}}{2}\right)\cos\alpha \right)\right\}}^{2}+{\left(x-h\sin\alpha -\frac{l_{A}}{2}\sin\alpha \right)}^{2}\sin^{2}\alpha +w^{2}{\}}^{3/2}$$

(20) $$\gamma_{6}=\{{\left\{-\left[d+\frac{l_{D}}{2}\right]-\left(l-\left(l-h-\frac{l_{A}}{2}\right)\cos\alpha \right)\right\}}^{2}+{\left(x-h\sin\alpha -\frac{l_{A}}{2}\sin\alpha \right)}^{2}\sin^{2}\alpha +w^{2}{\}}^{3/2}$$

(21) $$\gamma_{7}=\{{\left\{-\left[d-\frac{l_{D}}{2}\right]-\left(l-\left(l-h+\frac{l_{A}}{2}\right)\cos\alpha \right)\right\}}^{2}+{\left(x-h\sin\alpha +\frac{l_{A}}{2}\sin\alpha \right)}^{2}\sin^{2}\alpha +w^{2}{\}}^{3/2}$$

(22) $$\gamma_{8}=\{{\left\{-\left[d+\frac{l_{D}}{2}\right]-\left(l-\left(l-h+\frac{l_{A}}{2}\right)\cos\alpha \right)\right\}}^{2}+{\left(x-h\sin\alpha +\frac{l_{A}}{2}\sin\alpha \right)}^{2}\sin^{2}\alpha +w^{2}{\}}^{3/2}$$

where *w* is the distance between magnets A and B in the *z*-direction, which can be observed in Figure 3. By substituting equations (13) and (18) into equation (1), the total restoring force can be obtained.

## 4. Experimental validation

**Figure 6.**
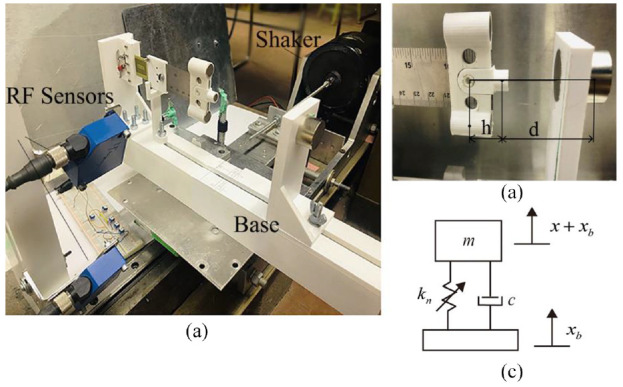
(a) Photo of the experimental setup, (b) detail of the beam, and (c) schematic of the equivalent lumped parameter model for the experimental setup.

With the models established, a natural question arises regarding their accuracy and reliability. To this end, an experimental model validation is conducted. For simplicity, hereinafter, the equivalent magnetic point dipole model and 2-point dipole model are referred to as first model and second model, respectively. The restoring force surface method (Worden, 1990) is employed to determine the total restoring forces dynamically. Figure 6(a) and (b) show the experimental setup and the detail of the magnets’ positions, respectively. Figure 6(c) shows a schematic of the equivalent lumped parameter model that represents the experimental setup, where $x_{b}$ is the base’s displacement and x is the displacement of the cantilever beam’s tip relative to the base, m represents the total mass of the assembly of magnets A and C and magnet B, c is the damping coefficient, and $k_{n}$ represents the stiffness of the combined spring. The lumped parameter model is commonly employed for multi-stable energy harvesters (Leng et al., 2017). The equation of motion of this setup is given by:

(23) $$m\left(\overset{\cdot \cdot }{x}+{\overset{\cdot \cdot }{x}}_{b}\right)+c\overset{\cdot }{x}+f_{x}\left(x\right)=0$$

where $f_{x}(x)$ denotes the restoring force of the combined spring. Equation (23) can be rewritten as:

(24) $$F\left(x,\overset{\cdot }{x}\right)=-m\left(\overset{\cdot \cdot }{x}+{\overset{\cdot \cdot }{x}}_{b}\right)$$

where $\;F(x,\overset{\cdot }{x})$ is the so-called restoring force surface.

As shown in Figure 6(a), the apparatus is mounted on a slipping table that is driven by a shaker (2809, Brüel & Kjær) through a stinger. The shaker is driven by an amplifier (2718, Brüel & Kjær). Two laser reflex sensors (RF) (CP24MHT80, Wenglor) are used to measure the transverse displacement of the beam’s tip and the base’s displacement, respectively. A computer equipped with the dSPACE dS1104 data acquisition board is used to collect sensor data and send voltage signal to the power amplifier to drive the shaker. The control program is developed by using the MATLAB Simulink which is interfaced with dSPACE Controldesk Desktop software. The velocity and acceleration are obtained by numerical differentiation of the measured displacement signals.

**Figure 7.**
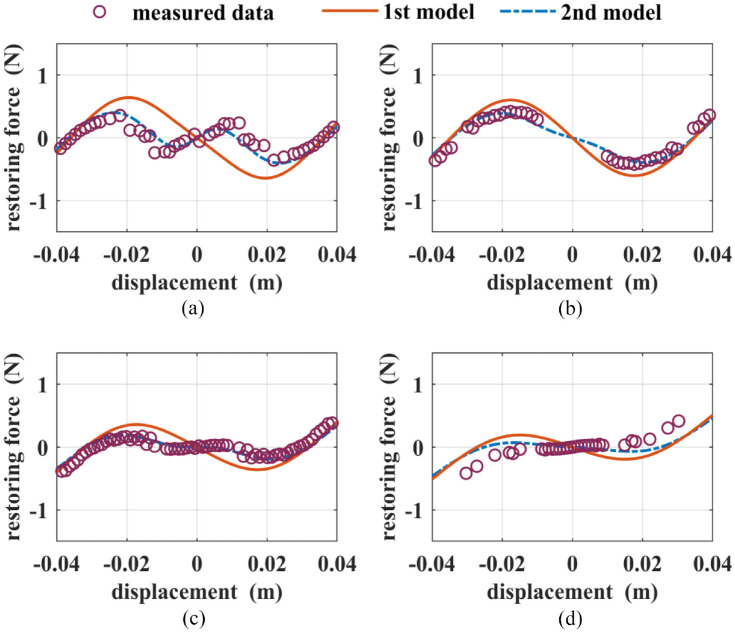
The total restoring forces of: (a) Case (1), (b) Case (2), (c) Case (3), and (d) Case (4).

To apply the restoring force method properly, the responses should sufficiently cover the phase plane. The exciting signal should be persistently strong so that both intrawell and interwell responses are established. For this purpose, a harmonic signal with a slowly modulated amplitude is employed

(25) $$x_{b}(t)=X_{b}\cos(0.2\pi t)\times \cos(2\pi f_{n}t)$$

where $X_{b}$ and $f_{n}$ are the amplitude and exciting frequency, respectively. The general guidelines for choosing proper values of $X_{b}$ and $f_{n}$ are that $X_{b}$ should be large enough to achieve interwell responses and $f_{n}$ should be close to the natural frequency of the linearized system around the equilibrium position. In the experiment, $X_{b}$ and $f_{n}$ are chosen on a case-by-case basis by trial and error. A great number of experiments are conducted to examine the relationship between the stability states and the tuning parameters. For the sake of comparison, the following four configurations are chosen: Case (1) *d* = 0.0407 m, *h* = 0.0187 m; Case (2) *d* = 0.0457 m, *h* = 0.0162 m; Case (3) *d* = 0.0517 m, *h* = 0.0187 m; Case (4) *d* = 0.0507 m, *h* = 0.0187 m. The purple circles in Figure 7 show the identified restoring force values. As shown in the figures, the system is transferred from a tri-stable one in Case (1) to a mono-stable one in Case (4). By using the parameter values given in Table 1 in the derived models, the analytical restoring forces are found. The blue dashed lines and red solid lines in Figure 7 show the restoring force values based on the first model and the second model, respectively. It can be seen that the first model fails to predict Cases (1), (3), and (4). On the other hand, the second model shows a better agreement with the measured data for Cases (1), (2), and (3) than the first model. But it fails to predict both the magnitude and trend for Case (4).

**Table 1.** Parameter values of the apparatus (Li, 2021).

Symbol	Description	Value
$V_{A}$ , $V_{C}$ ( $m^{3}$ )	Volume of magnet A and C	$3.21\times 10^{-6}$
$V_{B}$ ( $m^{3}$ )	Volume of magnet B	$3.93\times 10^{-7}$
$V_{D}$ ( $m^{3}$ )	Volume of magnet D	$1.29\times 10^{-5}$
$S_{A}$ , $S_{C}$ ( $m^{2}$ )	End surface area of magnet A and C	$3.22\times 10^{-5}$
$S_{B}$ ( $m^{2}$ )	End surface area of magnet B	$3.93\times 10^{-5}$
$S_{D}$ ( $m^{2}$ )	End surface area of magnet D	$1.29\times 10^{-5}$
$k_{b}$ (N/m)	Stiffness of the cantilever beam	26.17
*l* (m)	Length of the cantilever beam	0.12
*m* (kg)	Mass of the system	0.086
*c* (Ns/m)	Damping coefficient	0.0668

## 5. Model optimization

**Figure 8.**
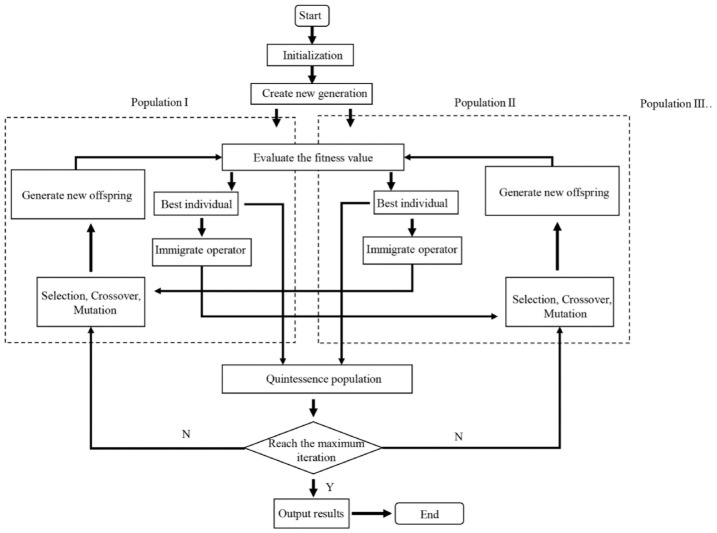
The flowchart of the MPGA.

As shown in the previous section, although the second model gives a better prediction for the restoring force than the first model in Cases (1), (2), and (3), it fails to do so in Case (4). It is natural to ask a question of whether both models can be improved by optimization. For this purpose, an optimization based on the multi-population genetic algorithm (MPGA) (Kim and Khosla, 1992) is carried out to identify the magnitudes of the magnetic vectors for the first model, and the amounts of the total charges for the second model. Different from the standard genetic algorithm, which only has a single population group, the MPGA initializes the whole population as multiple population groups to operate the selection, crossover, and mutation independently. Figure 8 shows the flowchart of the MPGA. Note that the flowchart only shows two population groups as an example. In the beginning, the initial ranges of the parameters, the population size, the population group number, and the maximum iteration number need to be specified. After the initialization, the individuals of the first population are randomly generated within the specified ranges, and they are arranged into different population groups. Then, the fitness values or objective functions are evaluated. The best individual of each population group will immigrate to the other population groups and participate in the respective groups’ selection, crossover, and mutation operation process. The main purpose of the immigration operator is to prevent the decrease in genetic diversity of a single population group. After that, the new offspring will be generated and prepared for the evaluation process in the next iteration. On the other hand, the best individual of each iteration will always be collected to the quintessence population group. As the maximum iteration number is reached, the individual in the quintessence population who has the minimum fitness value will be chosen as the optimum individual.

The training data of the optimization are chosen from the measured restoring forces of the three different configurations of the system: Case (5) *d* = 0.0587 m, *h* = 0.0187 m; Case (6) *d* = 0.0502 m, *h* = 0.0162 m; Case (7) *d* = 0.0372 m, *h* = 0.0162 m, which make the system exhibit mono-stable, bi-stable, and tri-stable stability state, respectively. The main reason for choosing the configuration cases different from Cases (1) to (4) is to prevent the local minima problem to happen in optimization. In this way, it will guarantee that the optimized model is able to predict any configuration in the system’s parameters region. The parameters to be optimized for the first model are chosen to be $m_{A}$ , $m_{B}$ , and $m_{D}$ . First, to have a fair comparison, the parameters to be optimized for the second model are chosen to be $Q_{A}$ , $Q_{B}$ , and $Q_{D}$ where $Q_{1}=-Q_{B}$ , $Q_{2}=Q_{B}$ , $Q_{3}=-Q_{A}$ , $Q_{4}=Q_{A}$ , $Q_{5}=-Q_{D}$ , $Q_{6}=Q_{D}$ . The fitness function used in the optimization for the first model is defined as:

(26) $$J_{1}(m_{A},m_{B},m_{D})=\sqrt{\frac{1}{3N}\sum_{j=5}^{7}\sum_{i=1}^{N}{\left(f_{\mathrm{jm}}(x_{i})-f_{\mathrm{ja}1}(x_{i})\right)}^{2}}$$

and the fitness function used in the optimization for the second model is defined as

(27) $$J_{2}(Q_{A},Q_{B},Q_{D})=\sqrt{\frac{1}{3N}\sum_{j=5}^{7}\sum_{i=1}^{N}{\left(f_{\mathrm{jm}}(x_{i})-f_{\mathrm{ja}2}(x_{i})\right)}^{2}}$$

where $f_{\mathrm{jm}}(x_{i})$ is the measured restoring forces that are smoothened by a spline fitting, $f_{a1}(x_{i})$ is the analytical restoring forces based on the first model, $f_{a2}(x_{i})$ is the analytical restoring forces based on the second model, and N=81 is the number of data. The reasons for interpolating the measured restoring forces with spline fitting are twofold: to alleviate the influence of measurement noise and to use the same amount of data in optimization for all cases that have different numbers of the raw data. The parameter search ranges for the first model are chosen as $0\leq m_{A}\leq 10$ , $0\leq m_{B}\leq 1$ , and $0\leq m_{D}\leq 30$ , and the parameter search ranges for the second model are chosen as $0\leq Q_{A}\leq 400$ , $0\leq Q_{B}\leq 200$ , and $0\leq Q_{D}\leq 1200$ . For both models, the maximum number of iterations is set to be 200, and the number of the population group and the size of each group are set to 100 and 500, respectively.

Table 2 lists the optimization results where the differences between the original values and the optimized values are represented by σ . It can be seen that the original magnitudes of the magnetic vectors are underestimated for $m_{A}$ and $m_{B}$ and overestimated for $m_{D}$ . And there is a significant difference between the original magnitude and optimized magnitude for $m_{B}$ . On the other hand, for the second model, the original amounts of the total charges are underestimated for $Q_{B}$ and $Q_{A}$ and overestimated for $\;Q_{D}$ . Accordingly, for both modeling approaches, the effect of magnet B is underestimated while the effect of magnet D is overestimated, which is the leading cause of the errors in prediction, as shown in Figure 7.

**Table 2.** The optimization results of the first model and second model.

	First model		σ %	Second model with three independent parameters		σ %
	Original	Optimized		Original	Optimized	
Magnet A	$m_{A}=3.74$	$m_{A}^{*}=3.802$	1.6	$Q_{A}=147.17$	$Q_{A}^{*}=209.14$	42.10
Magnet B	$m_{B}=0.46$	$m_{B}^{*}=0.803$	74.56	$Q_{B}=57.49$	$Q_{B}^{*}=80.13$	39.38
Magnet D	$m_{D}=14.95$	$m_{D}^{*}$ = 11.96	−20	$Q_{D}=588.71$	$Q_{D}^{*}=334.78$	−43.13

**Figure 9.**
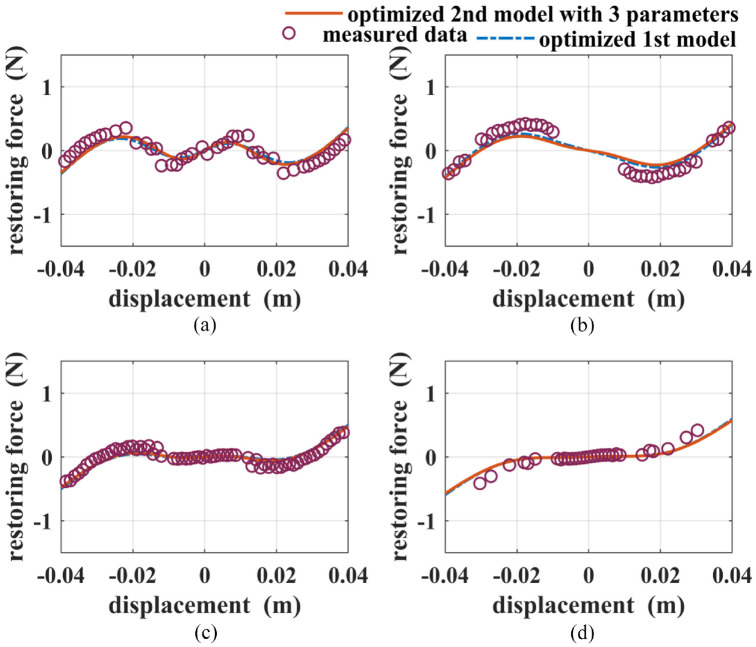
The total restoring forces for: (a) Case (1), (b) Case (2), (c) Case (3), and (d) Case (4) based on the optimized models.

Using the optimum parameters, the simulations of the restoring forces for Cases (1), (2), (3), and (4) are conducted, and the results are shown in Figure 9. The blue dashed lines and red solid lines represent the values of the restoring force based on the optimized first model and the optimized second model, respectively. It can be seen that both optimized models fit the measured values well for all four cases. Table 3 gives a quantitative comparison of the fitness values for the four cases. It can be seen that the fitness value for the first model is drastically reduced and becomes slightly smaller than the fitness value for the second model. Clearly, the proposed optimization significantly improves the accuracy of the first model.

**Table 3.** The fitness value of Case (I) to (IV) using original and optimized first and second model.

	Model categories	Fitness values
Original model	Original first model	0.248
	Original second model	0.131
Optimized model	Optimized first model	0.104
	Optimized second model with three independent parameters	0.111
	Optimized second model with six independent parameters	0.0919
	Optimized second model with five independent parameters	0.0917

**Figure 10.**
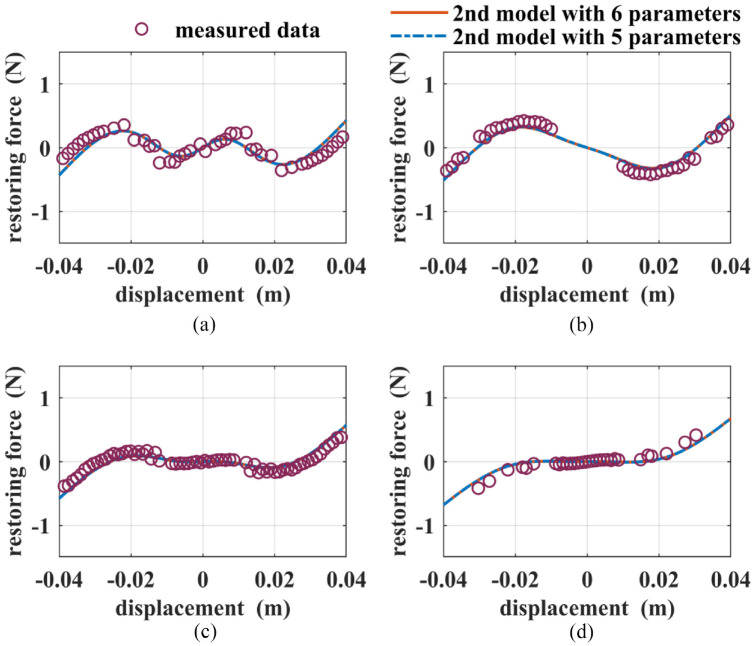
The total restoring forces for: (a) Case (1), (b) Case (2), (c) Case (3), and (d) Case (4) based on the optimized second models with six and five independent parameters.

The above optimization indicates that both optimum models offer a comparable accuracy for the prediction of the restoring forces. As the second approach treats a magnet as a 2-point dipole, it provides more freedom for controlling the model accuracy. One of the possible ways to further improve the model accuracy is to consider all the total charges $Q_{1}$ , $Q_{2}$ , $Q_{3}$ , $Q_{4}$ , $Q_{5}$ , and $Q_{6}$ as independent parameters. A six-parameter optimization is conducted by using the same parameter ranges and initialization process mentioned above. Note that the fitness function in equation (27) now becomes $J_{2}(Q_{1},Q_{2},Q_{3},Q_{4},Q_{5},Q_{6})$ . The results are given in Tables 3 and 4. Table 3 shows that the accuracy model can be further improved if all the total charges are identified. And as shown in Table 4, the almost zero value for the optimum charge $Q_{3}^{*}$ warrants an investigation. A plausible explanation is that, as shown in Figure 5, the left surface of magnets A and C is farthest away from magnet D, which means the effect of this surface is less critical in the magnetic force model. Thus, an assumption can be made that the total charge $Q_{3}$ can be neglected so that there are five independent parameters to be optimized. By defining the fitness function as $J_{2}(Q_{1},Q_{2},Q_{4},Q_{5},Q_{6})$ , a five-parameter optimization is conducted. The results are shown in Tables 3 and 4 as well. It can be seen that the fitness values for the second models with six and five independent parameters are almost the same. After conducting simulations for Cases (1) to (4) based on such two models, the results are shown in Figure 10. The red solid lines and the blue dashed lines in Figure 10 represent the values of the restoring force based on the optimized second model with six and five independent parameters, respectively. It’s found that both two models fit the measured ones well for all four cases and their predicted values are almost the same. The results validate the assumption that the total charge $Q_{3}$ of the second model can be neglected in the optimization, and it also proves that the simplified five-parameter optimization can make the second model reaches the same accuracy level as the optimum six-parameter model does.

**Table 4.** The optimization results of the second model with six or five parameters.

	Second model with six independent parameters		σ %	Second model with five independent parameters		σ %
	Original	Optimized		Original	Optimized	
Magnet A	$Q_{3}=147.17$	$Q_{3}^{*}=0.0017$	−99.99	$Q_{3}=147.17$	$Q_{3}^{*}=0$	−100
	$Q_{4}=147.17$	$Q_{4}^{*}=227.05$	54.27	$Q_{4}=147.17$	$Q_{4}^{*}=295.67$	100.9
Magnet B	$Q_{1}=57.49$	$Q_{1}^{*}=13.15$	−77.13	$Q_{1}=57.49$	$Q_{1}^{*}=11.96$	−79.19
	$Q_{2}=57.49$	$Q_{2}^{*}=55.95$	−2.68	$Q_{2}=57.49$	$Q_{2}^{*}=70.72$	23.01
Magnet D	$Q_{5}=588.71$	$Q_{5}^{*}=497.74$	−15.45	$Q_{5}=588.71$	$Q_{5}^{*}=388.73$	−33.96
	$Q_{6}=588.71$	$Q_{6}^{*}=892.90$	51.67	$Q_{6}=588.71$	$Q_{6}^{*}=698.95$	18.72

## 6. The parametric sensitivity study and stability state region

The sensitivity study intends to evaluate the model robustness against parametric variation. For this purpose, each of the three parameters in the optimum first model and the optimum second model with three parameters is perturbated by ±10% . The error between the optimum first model and perturbated first model is defined as:

(28) $$e_{j}=\sqrt{\frac{1}{N}\sum_{i=1}^{N}{\left(f_{\mathrm{ja}1}^{P}(x_{i})-f_{\mathrm{ja}1}^{*}(x_{i})\right)}^{2}}\;j=5,6,7$$

where $f_{\mathrm{ja}1}^{\;*}(x_{i})$ and $f_{\mathrm{ja}1}^{\;P}(x_{i})$ are the restoring forces of the optimum first model and the perturbated first model, respectively, N=81 is the number of the data used. The error between the optimum second model and the perturbated second model is defined as:

(29) $$e_{j}=\sqrt{\frac{1}{N}\sum_{i=1}^{N}{\left(f_{\mathrm{ja}2}^{P}(x_{i})-f_{\mathrm{ja}2}^{*}(x_{i})\right)}^{2}}\;j=5,6,7$$

where $f_{\mathrm{ja}2}^{\;*}(x_{i})$ and $f_{\mathrm{ja}2}^{\;P}(x_{i})$ are the restoring forces of the optimum second model and the perturbated second model, respectively. Table 5 lists the errors by perturbating one parameter by 10%. It should be noted that if the parameter is perturbated by −10%, the errors remain unchanged. Based on the results, several observations can be drawn for both models. The parameter variation of magnet A and C affects the restoring forces most significantly. The tri-stable state is most sensitive to the parameter variation while the mono-stable state is least sensitive to the parameter variation. In addition, based on the average errors given in the last column of Table 5, the second model is slightly more robust than the first model when the parameters of magnets A (C) and B are perturbated, and both models have equal robustness when the parameter of magnet D is perturbated.

**Table 5.** Errors for Cases (5), (6), and (7) of the optimum first and second models with detuned parameters.

Model	Parameters	$e_{5}$	$e_{6}$	$e_{7}$	$\bar{e}=\frac{e_{5}+e_{6}+e_{7}}{3}$
		Mono-stable	Tri-stable	Bi-stable	
1	$1.1m_{A}^{*},\;m_{B}^{*},\;m_{D}^{*}$	$5.2\times 10^{-2}$	$16.0\times 10^{-2}$	$9.0\times 10^{-2}$	$10.1\times 10^{-2}$
	$m_{A}^{*},\;1.1m_{B}^{*},\;m_{D}^{*}$	$2.0\times 10^{-2}$	$10.9\times 10^{-2}$	$3.6\times 10^{-2}$	$5.5\times 10^{-2}$
	$m_{A}^{*},\;m_{B}^{*},1.1m_{D}^{*}$	$3.2\times 10^{-2}$	$7.0\times 10^{-2}$	$5.5\times 10^{-2}$	$5.2\times 10^{-2}$
2	$1.1Q_{A}^{*},\;Q_{B}^{*},\;Q_{D}^{*}$	$4.2\times 10^{-2}$	$11.9\times 10^{-2}$	$7.1\times 10^{-2}$	$7.7\times 10^{-2}$
	$Q_{A}^{*},\;1.1Q_{B}^{*},\;Q_{D}^{*}$	$1.0\times 10^{-2}$	$6.9\times 10^{-2}$	$2.0\times 10^{-2}$	$3.3\times 10^{-2}$
	$Q_{A}^{*},\;Q_{B}^{*},\;1.1Q_{D}^{*}$	$3.2\times 10^{-2}$	$7.1\times 10^{-2}$	$5.2\times 10^{-2}$	$5.2\times 10^{-2}$

**Figure 11.**
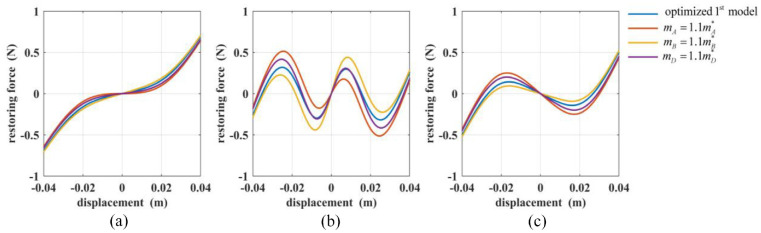
The total restoring forces of the optimized and perturbated first model for: (a) Case (5), (b) Case (6), and (c) Case (7).

**Figure 12.**
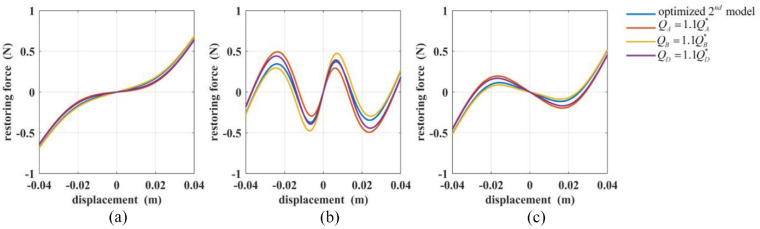
The total restoring forces of the optimized and perturbated second model for: (a) Case (5), (b) Case (6), and (c) Case (7).

Figures 11 and 12 shows the restoring forces values based on the optimum models and perturbated model. The blue lines represent the restoring forces of the optimum first model or second model, and the red, yellow, and purple lines are the restoring forces of both models when varying the parameters of magnet A, magnet B, and magnet D, respectively. The figures confirm the observations made above. The figures also show the effect of the parameter variation on the trends of the restoring forces. As shown in Figures 11(a), (b) and 12(a), (b), an increase in $m_{B}$ or $Q_{B}$ strengthens the mono-stable or tri-stable state most significantly while an increase of $m_{A}$ or $Q_{A}$ weakens the mono-stable or tri-stable state most significantly. As shown in Figures 11(c) and 12(c), an increase of $m_{A}$ or $Q_{A}$ results in a stronger bi-stable state while an increase of $m_{B}$ or $Q_{B}$ results in a weaker bi-stable state. Such effects are expected as magnet B is critical for the mono-stable state or tri-stable state while magnets A and C are critical for the bi-stable state. The opposite effects occur when the parameters are decreased by 10%.

**Figure 13.**
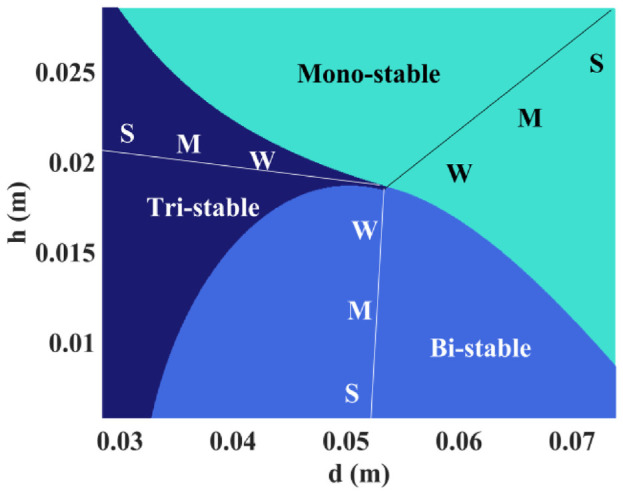
Stability state region.

With the optimum models, the so-called stability state region can be generated by varying the tuning parameters d and h . Figure 13 shows such plot by using the optimum second model with five independent parameters, where S, M, and W denote the strong, medium, and weak stability state based on the depth of potential wells, respectively. The stability state region clearly shows that by tuning d and h , the proposed apparatus can achieve the tri-stable, bi-stable, and mono-stable stability states, respectively.

## 7. Conclusions

In this study, a tunable multi-stable piezoelectric energy harvester has been developed for applications in an ambient vibration environment with a broad frequency band. The apparatus can be manually tuned to achieve tri-stable, bi-stable, and mono-stable stability states. The magnetic restoring forces of the apparatus have been derived by using two approaches named as first model and second model, respectively. An experimental validation of both models has been conducted. It has been found that although the second model is more accurate than the first model, it has its own limitation. A model optimization has been carried out by using the multi-population genetic algorithm (MPGA). The magnitudes of the magnetic vectors and the amounts of the surface charges of the three magnets have been chosen as parameters to be optimized for the first and second model, respectively. The results show that two optimum models can achieve almost the same level of accuracy. The results also show that the optimum second model has a larger error in predicting the restoring force of the bi-stable state case than the optimum first model. To further improve the accuracy of the second model, the six-parameter optimization has been carried out by assuming that the two surface charges of an individual magnet are different. The results show that the accuracy of the second model with six independent parameters can be further improved. The results also show that the optimum value of $Q_{3}$ is almost zero, as the corresponding surface is farthest away from magnet D. Based on this observation, magnets A and C can be treated as one point dipole so that the number of independent parameters can be reduced to five in the optimization. The results show that the optimum second model with five parameters has the highest accuracy among all the three optimum models. With the optimum models, the parametric sensitivity has been investigated by perturbating each of the three parameters by ±10% . The following observations have been drawn. The parameter variation of magnet A affects the restoring forces most significantly. The tri-stable state is most sensitive to the parameter variation, while the mono-stable state is least sensitive to the parameter variation. In addition, the second model is slightly more robust than the first model when the parameters of magnets A and B are perturbated, and both models have equal robustness when the parameter of magnet D is perturbated. With the optimum second model, the stability state region has been generated to show that the developed apparatus possesses a large parameter tuning space.
